# Exosomal Long Noncoding RNAs: Insights into Emerging Diagnostic and Therapeutic Applications in Lung Cancer

**DOI:** 10.1155/2020/7630197

**Published:** 2020-11-10

**Authors:** Mohammad Reza Karimzadeh, Mohammad Reza Seyedtaghia, Mohammad Soudyab, Maria Nezamnia, Jason Kidde, Amirhossein Sahebkar

**Affiliations:** ^1^Department of Medical Genetics, School of Medicine, Bam University of Medical Sciences, Bam, Iran; ^2^Department of Medical Genetics, School of Medicine, Mashhad University of Medical Sciences, Mashhad, Iran; ^3^Department of Obstetrics and Gynecology, School of Medicine, Bam University of Medical Sciences, Bam, Iran; ^4^Department of Emergency Medicine, University of Utah, Salt Lake City, UT, USA; ^5^Biotechnology Research Center, Pharmaceutical Technology Institute, Mashhad University of Medical Sciences, Mashhad, Iran; ^6^Neurogenic Inflammation Research Center, Mashhad University of Medical Sciences, Mashhad, Iran; ^7^Halal Research Center of IRI, FDA, Tehran, Iran

## Abstract

Lung cancer is the most common cause of cancer-related deaths worldwide. Annually, millions of people die from lung cancer because of late detection and ineffective therapies. Recently, exosomes have been introduced as new therapeutic players with the potential to improve upon current diagnostic and treatment options. Exosomes are small membranous vesicles produced during endosomal merging. This allows for cell packaging of nucleic acids, proteins, and lipids and transfer to adjacent or distant cells. While exosomes are a part of normal intercellular signaling, they also allow malignant cells to transfer oncogenic material leading to tumor spread and metastasis. Exosomes are an interesting field of discovery for biomarkers and therapeutic targets. Among exosomal materials, lncRNAs have priority; lncRNAs are a class of noncoding RNAs longer than 200 base pairs. In the case of cancer, primary interest regards their oncogene and tumor suppressor functions. In this review, the advantages of exosomal lncRNAs as biomarkers and therapeutic targets will be discussed in addition to reviewing studies of their application in lung cancer.

## 1. Introduction

Lung cancer is the most prevalent cancer in men and is the third most common cancer in women following breast and coloncancers [1]. In 2018 alone, there were 2 million lung cancer diagnoses [1]. Smoking is the most prominent risk factor for lung cancer, which explains the discrepancy between men and women, second to the higher proportion of male smokers [2]. The age-standardized mortality rate for lung cancer is 19.7 in 100,000 relative to an incidence of 23.1 in 100,000 [3], making it the most lethal of all cancers. Its lethality is further illustrated by a five-year overall survival of 10–15%.

Lung cancer is classified into nonsmall cell lung cancer (NSCLC) and small cell lung cancer (SCLC), comprising 85% and 15% of cases, respectively. NSCLC is further subdivided into adenocarcinoma, squamous cell carcinoma, large cell carcinoma, and undifferentiated nonsmall cell lung carcinoma [4].

There is often a delay in diagnosis of lung cancer second to nonspecific symptoms, resulting in late-stage diagnosis and poor prognosis. Additionally, routine tests such as chest x-rays have low sensitivity, and confirmatory tests such as sputum cytology and lung biopsy require a high index of suspicion based on abnormal imaging results [5]. Therefore, there is a clear need for more reliable screening and diagnostic markers.

Exosomes are small saucer-like membranous extracellular vesicles that participate in intercellular signaling and are found in various human fluids including, but not limited to, blood [6], urine [7], and salvia in varying concentrations [8]. They originate from late endosomes differing from plasma membrane-originated vesicles (i.e., microvesicles) [9, 10] and are released from normal cells [11], apoptotic cells [12], and from cancerous cells in high quantities [13]. The endosomal system is the starting point of exosome formation, which progresses toward the extracellular transport by the way of ESCRT (the endosomal sorting complexes required for transport) machinery [14], although there is evidence of ESCRT-independent mechanisms via tetraspanin proteins as well [15, 16]. They are the smallest (30–150 nm) [17] extracellular vesicles (EVs) as compared to microvesicles, which are 100 nm up to 1000 nm diameter [10], and are further defined by their biogenesis, contents, and function [18–20].

The type and quantities of nucleic acid, proteins, lipids, and sugars within the exosome are specific to the parent cell [21]. This nonidentical exosome material is a promising feature, and in that, it makes analysis of exosomes a worthwhile exploratory target for new biomarkers and especially tumor markers [22]. While ultracentrifugation can isolate exosomes from other contents via variations in density [23], this process is laborious and time consuming [24]. Alternatively, isolating nucleic acids within the exosome are more specific and targetable. Specifically, both DNA (genomic and mitochondrial [25]) and to a greater degree, RNA species are present in exosomes [18]. Regarding cancer, transfer of carcinogenic exosomal RNA's in exosomes can affect all steps in the metastatic process [26]; however, this same mechanism of pathogenesis may be an exploitable fingerprint.

For many years, scientists have mostly investigated mRNAs regarding cancer; however, we now know that only 2% of our genome is protein-coding, and that mRNA consists of 1–5% of a cell's transcriptome, while probably more than 90% of the human genome is transcribed, and in some regions, transcription is bidirectional [27]. Most of the human transcriptome is noncoding RNA (ncRNA). ncRNAs perform various cellular functions including (1) protein synthesis (tRNA/rRNA), (2) protein export (7sl RNA), (3) RNA maturation, (4) DNA synthesis (Y RNA and RNase MRP), (5) telomer function [28], (6) transposon control [29], and (7) gene regulation.

Long noncoding RNAs (lncRNAs) are a group of ncRNAs with more than a 200 bp length transcript without an open reading frame [30]. Subsequently, many transcripts overlap with coding gene loci (partly or completely) [30]. Three types of lncRNAs exist: the first group is transcribed from the sense strand of a gene (named antisense RNA), not spliced, and functionally downregulates overlapping genes; the second group is transcribed from antisense DNA (named sense RNA), spliced like protein-coding genes, and regulates adjacent genes including genes on distant chromosomes in some cases; and the third group is intronic lncRNAs [30]. lncRNAs have both oncogenic and tumor-suppressing roles [31] and contribute to the progression of metastasis [32] and reprogramming metabolism in cancer cells to foster survival in substrate-limited or acidic microenvironments [33]. Importantly, lncRNAs are highly represented in exosomes.

While up to 270,044 lncRNA genes exist [34] so far, only 200 lncRNAs have been studied in depth. lncRNAs are likely to be paramount to future cancer treatment [35] and diagnosis [36].

## 2. lncRNAs and Their Potential Roles in Cancers

For many years, the central dogma has been that genes are transcribed to mRNA and translated to protein, with the majority of the genome being nonfunctional (called junk DNA). We have since learned that not only the rest of the genome is important but also it can be transcribed from both strands with the exception of constitutional heterochromatin regions. The current opinion of transcriptomics is owed to advances in next-generation sequencing techniques that show various types of RNAs, which were previously neglected [37].

RNAs are classified into coding RNAs (also known as mRNAs) and noncoding RNAs. While the estimated number of coding genes has decreased, many thousands of noncoding genes (or RNAs) are being identified. A major portion of the transcriptome consists of rRNAs and tRNAs, which are the best-known ncRNAs with crucial roles in protein synthesis. Furthermore, categorization by size separates ncRNAs into those shorter than 200 nucleotides (miRNA, siRNA, snRNA, and snoRNA) and those between 200 and 100,000 nucleotides, which are classified as long noncoding RNAs [38]. The last number of lncRNA genes estimation was 270,044 [34].

lncRNAs are difficult to classify because of the wide variety of size, diverse location in the genome, and numerous functions in the cell [39]. Regarding classification by genome location, lncRNAs consists of two subclasses. The first is long intergenic noncoding RNAs (lncRNAs) that could be further classified into sense and antisense. The second is long intronic noncoding RNAs [40]. lncRNAs are structurally similar to mRNAs because they have 5′cap and poly-A tail and also could be spliced [41]. Some lncRNAs have a specific promoter, while some have a common promoter with coding RNAs and other ncRNAs [40]. Interestingly, some lncRNAs recruit enhancers instead of promoters [42]. lncRNAs usually are transcribed by RNA polymerase II but in some cases could be transcribed by RNA polymerase III because of the type of their promoters [43].

The roles of lncRNAs in some types of diseases such as neurodegenerative disorders, cardiovascular disorders, and several types of cancers have been reported [40]. Most lncRNAs are defined functionally by either gene upregulation or downregulation; however, despite their name, some have ORF and are translated to functional proteins as determined by ribo-seq and mass spectrometry. They are categorized into the following six functional groups: first, lncRNAs modulate signaling pathways by regulating protein expression [44]; second, decoy lncRNAs occupy decoy sites restricting transcription factors [45] and also act as miRNA sponges [46]; third, scaffold lncRNAs provide a medium for assembling and recruiting factors to make multicomponent complexes such as RNPs (ribonucleoproteins) observed in transcription and ubiquitination [47]; fourth, guide lncRNAs play roles in localization and direct TFs (transcription factors), proteins, and RNPs in a cis or trans site manner [48]; fifth, enhancer RNAs (eRNAs) facilitate chromatin looping to interact with enhancers and promoters [49]; and sixth and last class are lncRNAs with ORFs, which may produce functional peptides [50].

Evolving understanding of lncRNAs will likely lead to further identifying functions and classifications. Plausible roles in biological processes include epigenetics, splicing, stabilizing of mRNAs and proteins, and trafficking nuclear components.

lncRNAs have important roles in the formation of malignant cells, cancer progression, and metastasis by way of both oncogenic and tumor-suppressing activities. Regarding tumor-suppressing function, lncRNAs can promote apoptosis and oncogenic miRNA sponges, as well as arrest both the cell cycle and epidermal mesenchymal transition (EMT). For example, downregulation of MEG3 would suppress the growth of some types of cancer cells by inhibiting the Wnt pathway and also PTEN, while upregulating TP53 [51, 52]. Furthermore, MEG3 acts as an oncogenic miRNA sponge inhibiting the tumor suppression roles of miR-21 and miR-421 [53]. PANDAR is another example. This lncRNA could interact with BCL-2 leading to NSCLC apoptosis [54]. Interestingly, PANDAR has an oncogenic role in other types of cancers as well [55]. PANCR is another tumor suppressor lncRNA that regulates metastasis, possibly through the regulation of EMT in NSCLC. Patients with a low level of PANCR have a poor prognosis [56].

lncRNAs can regulate oncogenes, and dysregulation of this class of genes have been observed in inducing cell cycle progression, arresting apoptosis, multidrug resistance, cell migration and invasion, angiogenesis, epithelial EMT, and metastasis. The first example of oncogenic lncRNAs is SNHG1. Upregulation of SNHG1 is involved in the progression of cell growth in colorectal cancer [57]. This RNA directly interacts with PRC2 (polycomb repressive complex), which is involved in histone modification and leads to downregulation of KLF2 and CDKN2B. It also acts as an miR-154-5p sponge, which is a repressor of CCND2. Knockdown of SNHG1 significantly suppresses the growth of CRC (colorectal cancer) cells [57]. The second example is AFAP1-AS1, an antiapoptotic lncRNA, which is transcribed from the sense strand of the AFAP1 locus [58]. Downregulating this lncRNA via siRNA in esophageal adenocarcinoma OE-33 cell line leads to cell cycle arrest in the G2/M phase resulting in apoptosis [58, 59]. The third example is a multidrug resistance lncRNA, LINC00518, that is a miR-199a sponge within the cytoplasm [60]. miR-199a represses MRP1, which could be involved in anticancer drug resistance in some types of cancers such as breast cancer [60]. The fourth example is NEAT1, which has been shown to be overexpressed in glioblastoma cancer [61]. NEAT1 could act as a sponge of both miR-449b-5p and miR-132. This leads to overexpression of c-Met [61] and SOX2, respectively [62], and potentiates cell migration and invasion. MCM3AP-AS1 is the fifth example of oncogenic lncRNAs that promotes angiogenesis [63]. MCM3AP-AS1 is a sponge of miR-211, leading to the upregulation of proteins involved in angiogenesis (KLF5 and AGGF1), which are observed in glioblastoma cancer [63]. MALAT1 (or NEAT2) is the sixth example. This lncRNA promotes metastasis in lung cancer by regulating the expression of genes associated with metastasis [64]. The last example is ARNILA, which competes with SOX4 on sponging of miR-204 resulting in increased SOX4 and resultant EMT and metastasis in breast cancer [65].

**Figure 1:**
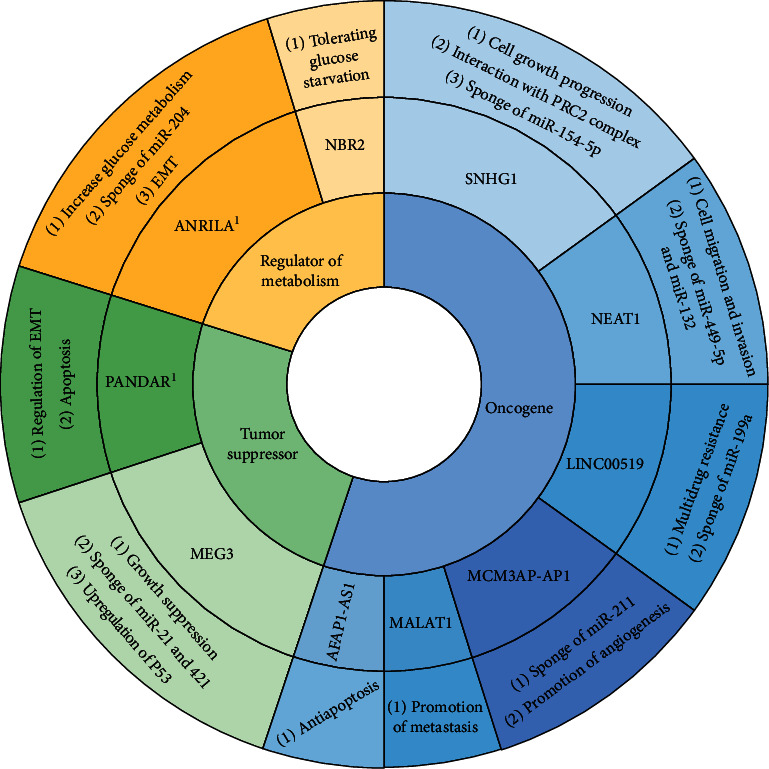
An overview of lncRNA roles in cancer as oncogenes, tumor suppressors, and regulators of metabolism.

lncRNAs have crucial roles in cancer metabolism, predominantly via increasing glycolysis, although they also influence metabolism of other substrates such as amino acids and lipids [66]. For example, ANRIL increases the expression of GULT1 and LDHA that leads to increase uptake and metabolism of glucose in nasopharyngeal carcinoma [67]. In another example, NBR2 helps cancer cells to tolerate glucose starvation [66]. lncRNA functions in cancer are summarily presented in Figure 1.

Given the involvement of lncRNA's in cancer, they make for intriguing pharmacologic targets. Additionally, their specificity to different types of cancers and concentrations in cancers vs normal tissues also make them viable diagnostic markers; however, knowledge of these molecules is growing and will certainly evolve.

## 3. Exosomes Function in Cancer Development

In the context of cell biology, there are three types of extracellular vesicles characterized by the size and mechanism of vesicle release: apoptotic bodies (bigger than 1000 nm), microvesicles (between 100 nm and 1000 nm), and exosomes (bigger than 30 nm and smaller than 150 nm) [17]. Exosomes are small bilayer lipidic saucer-like vesicles, which play an important role in cell-cell communication and homeostasis [68] via intercellular transfer of exosomal contents [9, 10, 69]. Recipient cells are targeted by surface adhesion macromolecules and tetraspanin complexes [70]. The fusion process is facilitated by the SNAREs (soluble N-ethylmaleimide-sensitive factor attachment protein receptor) complex and ESCRT machinery. ESCRT's machinery consists of four proteins (ESCRT type 0–III) and has vital roles in exosome synthesis [14].

Exosomes are found in a broad range of body fluids including blood, urine, [6, 7], breast milk [71], and bronchoalveolar lavage fluid [72]. They are a product of cell vesicular trafficking machinery and generally originate from late endosomes during fusion of two membranous particles [15]. There are three main stages of the biogenesis of exosome: first, the invagination of the plasma membrane creating an early endosome; second, budding from the endosome membrane forming multivesicular bodies (MVBs); and third, fusion of the late endosome to the plasma membrane and release of the MVBs [73].

Synthesis of exosomes is a regulated process, dependent on cell types, environmental stresses, and contact with other cells. For example, the amount of exosomes that are released by mesenchymal stem cells is significantly higher than immature dendritic cells, and in the context of environmental stress, hypoxia increases release of exosomes. Cells which have contact inhibition and cells that are in a quiescent phase have diminished exosome release [17, 74]. It is inferable that cells with higher division rates such as cancerous cells release more exosomes, which is in fact, has been shown to be the case [75].

Depending on the parent cell, exosome content differs by type and quantity. Exosomes consist of proteins, lipids, nucleic acids (including dsDNA), and other metabolites [76]. The contents within exosomes have crucial roles in normal cellular functions and in development of and responses to disease. Exosomes take a part in angiogenesis, metastasis, cell death, immune responses, inflammation, oncogenesis, and promotion of other diseases such as neurodegenerative disorders that include Parkinson's disease [77, 78]. Exosomes derived from cancer cells (also known as oncosomes) will be the focus.

The transition of a normal cell to a malignant cell is a multistep process consisting of initiation, promotion, and progression. Exosomes have been implicated in the promotion and progression of malignancy. Proteinic materials of oncosomes may facilitate normal cells and precancers to progress towards malignancy via mutated membrane proteins, signaling pathway intermediates (most cases), or transcription factors. This process could occur repeatedly between cells in the cancer milieu and accelerate carcinogenesis. Meanwhile, normal cell's exosomes transfer tumor-suppressing proteins (and also RNAs) that competitively halt carcinogenesis; however, carcinogenesis is favored because of the higher rate of exosomes (i.e., oncosomes) released by malignant cells.

This process is not limited to proteinic contents and could also be applied to nucleic acids in exosomes. Of the types of nucleic acids, lncRNAs and miRNAs have the most recognized regulatory roles. lncRNAs are discussed separately in the following sections.

In the case of metabolism reprogramming, exosomes from cancer-associated fibroblasts (CAFs) contain Krebs cycle intermediates, amino acids, and lipids. Some of these metabolites arrest mitochondrial oxidative metabolism and increase anaerobic glycolysis referred to the Warburg effect [79, 80]. CAFs are abundant in the cancer environment, and recipients of their exosomes are usually cancer cells [79].

Exosomes' effect on the immune response in cancer ranges from boosting the immune response or suppressing it, depending on the exosome's parent cell. Exosomes derived from the antigen-presenting cells contain surface antigen-presenting MHCs and lead to an exaggerated immune response. For example, dendritic cell-derived MHC^+^ exosomes in mice showed decreasing tumor size by triggering T cells. As such, animal models without functional T cells do not show decrease in tumor size [81]. Alternatively, tumor-derived exosomes can also result in evasion of immune surveillance. Prostate tumor-derived exosomes presenting Fas ligand induces T cell apoptosis, thereby decreasing T cell proliferation [82, 83]. Additionally, exosomes could modulate the innate immune system. We know that chronic inflammation (part of the innate immune system) participates in the development of the various cancer types [84]. The collaboration of exosomes in the inflammatory system (by their cargo, e.g., IL-6 and TNF*α*) fosters a protumor microenvironment and promotes cancer [84].

Exosomes contribute to metastasis (the main cause of death in cancer). Tumor-derived exosomes (TDEs) carry materials essential for EMT and metastasis such as TGF-*β* (inducer of EMT), HIF1-*α* (essential factor for angiogenesis), *β-*catenin (role in proliferation, angiogenesis, and migration) and MMPs (a group of protease which decoy extracellular matrix facilitating migration), which induce invasiveness and boost the capacity of cancer cell migration [85].

Another mechanism by which exosomes affect cancer is facilitating drug resistance. This phenotype could be induced in target cells by adding an ABC transporter (drug efflux pump) to their surface during merging or via some type of regulatory contents such as miRNAs or lncRNAs [86].

Exosomes play important roles in tumor microenvironment (TME) communication [87]. The tumor microenvironment (TME) is a complex multifaceted array of factors including the malignant cells, adjacent stromal cells, and functional characteristics of perfusion, inflammation, chemistry, and metabolism. These factors facilitate energy utilization, angiogenesis, and concealment from the immune system. The interaction of exosomes with the tumor milieu, activation of inflammation, immune system suppression, promotion of metastasis, and induction of MDR (multidrug resistance) represent the varied armament of exosomes in cancer development.

## 4. Advantages of Exosomes in Cancer, Diagnosis, Prognosis, and Therapy

In the above section, adverse effects of exosomes were investigated. In this section, the opportunities that lie ahead in exosome-based biomarkers and therapeutic targets will be discussed. Finding less invasive and more reliable markers for cancer diagnosis as well as appropriate targets for therapy are trending areas of cancer research. Delay in diagnosis is a major prognosticator of mortality in cancer and emphasizes the need of reliable biomarkers for early recognition. Additionally, the diverse functional roles of exosomes and lncRNAs make for multiple potential therapeutic targets. Established and novel areas of investigation such as exosomes or lncRNAs have a substantial potential to meet these needs. The potential of exosomes as biomarkers is discussed below.

Two important factors of a biomarker are sensitivity and specificity. Sensitivity and specificity are parameters mostly related to true-positive rate and true-negative rate, respectively. These values are important for calculating positive and negative predictive values [88]. An example of a currently used biomarker in ovarian cancer is CA-125. Despite its prolonged use, CA-125 has a low sensitivity of less than 50% in stage I ovarian cancer and 80% in advanced disease, corresponding with a false-negative rate of 50% and 20% in early and advanced cancers, respectively. Additionally, its specificity for cancer is low second to poor differentiation between ovarian cancer and benign gynecological diseases (i.e., high false-positive rate) [89]. This example demonstrates the need for new tumor markers.

Noninvasive methods should have priority over more invasive methods such as biopsy and sputum cytology being that these tests are costly, have associated risks, and are unpleasant and inconvenient for patients. Serum markers (proteins, carbohydrates, and lipids) have been unremarkable, so other options such as circulating tumor cell-based (CTC) and exosome-based markers have subsequently been investigated. Exosomes appear to be superior to CTCs which are very rare (500 in 7.5 ml blood besides 5 million nuclear cells present in each ml of blood), although the laborious process to isolate exosomes should be considered [90]. A major advantage of exosomes is their high specificity [90].

Early detection of cancer yields timely interventions (drugs and surgery) is a significant advantage for increasing overall survival and improving patient' quality of life. Exosomal survivin is a plausible exosome biomarker for early detection of cancer. Survivin is a protein member of inhibitor of apoptosis (IAP), which is consistently elevated in prostate tumor-derived exosomes in comparison with benign prostatic hyperplasia and healthy controls (i.e., good sensitivity and specificity) [91]. Survivin level was also high in relapsed patients after chemotherapy, so it would be a promising assay in minimal residual disease and follow-up surveillance [91].

Easier detection methods for inspecting genomic contents of exosomes such as PCR-based methods make them a better choice in comparison with proteins or carbohydrates. PCR-based methods are also cheaper and have a high throughput, making them feasible options for detecting a combination of two or more tumor markers to improve sensitivity and specificity. Among genomic contents, lncRNAs are an appropriate choice because they are long enough for easier primer designing compared with miRNAs; however, the most important advantage is their stability. Currently, dozens of lncRNAs are reported with potential biomarker properties (prognostic and diagnostic). For the first time in 2015, exosomal lncRNA was reported in breast cancer as a tumor marker by Xu et al. [92]. This study discovered lncRNA RP11-445H22.4 which is highly expressed in breast cancer patients with a specificity of 74% and 92% sensitivity. This marker was more correlated with ER^+^ and PR^+^ versus HER2^+^ status [92]. Therefore, RP11-445H22.4 not only has an application in cancer diagnosis but also prognosis [92]. Overall, exosomal lncRNAs have high sensitivity and specificity (about 70% up to 94%) and combining them with other lncRNAs and miRNAs could boost those ranges [93].

Exosomes could be a mediator for drug delivery. They are small enough to easily transfer through cells, including the blood-brain barrier. A bilayer lipid membrane construction enables them to easily fuse to targets and survive in body. Furthermore, exosomes are nontoxic and unlikely to trigger immunogenic responses. Perhaps, the most important characteristic is their ability to target specific organs and cells via surface proteins, such as integrin, reducing off-target side effects.

Despite the potential in cancer therapeutics, to date, the application of exosomes has been unremarkable. There is some promise, however, of exosomes being used for cell-free vaccines (i.e., making vaccines without cell culturing), which could be used to activate the immune system against cancers. Some exosomes could be antigen-presenting like dendritic-derived exosomes that could directly activate NK cells or cytotoxic T cells. This process could occur in different ways such as presenting tumor antigen to immune cells, transfer their MHC to tumor cells during fusion, or transfer antigenic peptides to other antigen-presenting cells [81, 94].

## 5. Potential of Exosomal lncRNAs as a Biomarker or Therapeutic Target in Lung Cancer

Any alteration in DNA that leads to the onset or progression of cancer manifests in RNA expression levels, so exosome analysis is providing real-time monitoring of patients that is highly important in fast progressive cancers.

### 5.1. Exosomal lncRNA in Lung Cancer Progression and Their Potency to Serve as a Biomarker

In the above section, we described important roles of lncRNA and exosome in cancer and their potency to serve as a biomarker. This section will focus on lncRNA as a component of exosomes in lung cancer. The physiologic functions of lncRNAs are instructive for selecting a marker or target because it would decrease accidental differential expression among cases and controls.

Tumor markers have important roles in the detection of cancer, staging, and drug response or as assays of minimal residual disease. Lung cancer is one of the most dangerous types of cancer and the leading cause of cancer-related death. Indeed, 25% of total cancer deaths is second to lung cancer [95]. This type of cancer is difficult to detect and presents with vague signs and symptoms in early stages. Additionally, these signs and symptoms overlap with other pulmonary diseases such as pneumonia creating susceptibility for misdiagnosis (e.g., continuous bloody cough, chest pain, or shortness of breath) [95, 96]. Moreover, lag time in diagnosis prognosticates metastatic features, high mortality, and more complicated management [96]. Therefore, there is a demand for a screening test for those more susceptible to lung cancer such as heavy smokers, people with high second-hand smoke-exposure, people living in large cities (because of air pollution), asbestos-exposed, and a family history of all subtypes of lung cancer.

Early detection and treatment of cancer increases patient survival. Some serum markers such as SCC (squamous carcinoma antigen) and CEA (carcinoembryonic antigen) have both low sensitivity and specificity. The current approach of detecting lung cancer includes imaging (computed tomography and X-ray), endoscopic biopsy, and sputum cytology [97]. Biopsy is a highly invasive and imposes risks (e.g., hemothorax, pneumothorax, and infection). Furthermore, it does not solve the problem of low sensitivity [97]. This dilemma should drive us to look for another way that is both sensitive and specific in early detection and less invasive than current practices.

After determining the presence of lung cancer, specifying subtype of cancer, staging, grading, and genetic testing are crucial for determining therapy. Cell type and gene mutations indicate phenotype and cancer sequelae. Based on histological classification and type of initial cell that developed to cancer, lung cancer divides into two major classes. Most of the patients (about 85%) have nonsmall cell lung cancer (NSCLC), which is further divided into three subtypes: squamous cell lung cancer (25%), adenocarcinoma (40%), and large cell (10%) [95]. 15% is small cell lung cancer (SCLC), which is divided into many subtypes.

Current approaches of therapy are surgical excision of the tumor followed by chemotherapy which is second line. Chemotherapy drugs such as gefitinib and erlotinib (kinase inhibitor) demonstrate good results in NSCLC patients with mutation(s) in EGFR, but resistance to these drugs is common in patients with end-stage disease [98, 99]. Additionally, many diverse side effects arise in patients consuming chemotherapy drugs. Other types of therapy such as radiation therapy and targeted therapy are performed in some cases [95].

Recently, investigations for new screening methods suggest exosome-based approaches, for reasons already discussed. We have reviewed studies that evaluated exosomal lncRNAs in lung cancer which are summarized in Table 1. The first is a 2017 study conducted by Zhang et al. in which exosomes were isolated with ExoQuick from 77 NSCLC patients and healthy controls. MALAT1 level was assayed by qRT-PCR, and the result showed an increased level of MALAT1 in NSCLC significantly associated with lymph node involvement and stage of the tumor. This lncRNA is associated with invasiveness and metastatic features and has a role in regulating the progression of cell cycle. An antiapoptotic role was also suggested in previous studies and were reconfirmed in this study by knocking out MALAT1 with shRNA. MALAT1 is proposed as a biomarker in prognosis and diagnosis of NSCLC and also a therapeutic target [100].

Second, a 2018 study by Zhang et al. suggested RP11-838N2.4 as a therapeutic target for end-stage NSCLC patients. RP11-838N2.4 was found in erlotinib resistant cell lines established from sensitive cell lines that were grafted to nude mice. This lncRNA was measured by qRT-PCR in 78 NSCLC patients who were resistant to therapy with erlotinib. Results showed upregulation in RP11-838N2.4, and this lncRNA may serve as a diagnostic marker [99].

The third is a 2018 study by Wu et al. which introduced lnc-MMP2-2 as a target for therapy and predictor for metastasis. Via bioinformatics analysis, this lncRNA is suspected to act as an enhancer that increases expression of matrix metalloprotease 2 in the TGF-*β* pathway. Increased expression of lnc-MMP2-2 was observed in exosomes obtained from two lung cancer cell lines A549 and HMVEC‐L by ultracentrifugation [101].

Lei et al. study is the fourth study on exosomal lncRNA. This study was performed on two NSCLC cell lines: HCC827 and HCC4006. These cell lines were grafted continuously in media with gefitinib to produce gefitinib resistance. Expression analysis in exosomes isolated by the ExoQuick kit from these two cell lines showed the upregulation level of lncRNA H19. This lncRNA is suspected to act in resistance to gefitinib and also serves as a therapeutic target in EGFR^+^ NSCLC patients [98].

In another 2019 study (fifth), Teng et al. introduced a new biomarker for LSCC. This study demonstrated different expression levels of SOX2-OT between 75 LSCC patients and 79 control individuals. SOX2-OT level significantly increased in LSCC patients and had reasonable sensitivity (76%) and specificity (73.17%). The level of SOX2-OT was also correlated with TNM (tumor, node, and metastasis) stage [102].

A 2019 study by Cheng et al. (sixth) investigated GAS5 (growth arrest-specific 5). First, they isolated exosomes from mice which were exposed to urethane and developed adenocarcinoma. Subsequently, they analyzed levels of GAS5 in A549, H1299, and 95D lung cancer cells and normal 16HBE lung cells [103] demonstrating that GAS5 was downregulated in cancer cells, and cells with overexpressed GAS5 resulted in inhibition of cancer proliferation and increased apoptosis. This study also showed that GAS5 downregulation leads to decreased PTEN and increases in the level of AKT and PI3K (via competition of miRNA), which are related to angiogenesis. Li et al. similarly isolated exosomes from 64 patients with NSCLC with a total exosome kit and demonstrated GAS5 levels correlated inversely with the size of tumor and TNM stage [97]. Cheng concluded that GAS5 could be a therapeutic target, while Li proposed that it could be used as a cancer marker [97, 103].

In 2019, Bai et al. introduced two lncRNAs, SLC9A3-AS1 and PCAT6. These lncRNAs had significantly higher expression levels in patients (32 samples) in comparison with healthy controls (30 samples). They developed a chip for a multiplex assay of SLC9A3-AS1, PCAT6, and GAPDH in single quantitative polymerase chain reactions that could be utilized for early detection of LSCC [96].

In 2019, Rao et al. elucidated the correlation between the downregulation of HAGLR and increased detection rate of CTC with poor prognosis in NSCLC. In this study, plasma exosomes of 40 NSCLC patients were isolated and expression level of HAGLR were compared with 8 healthy controls by qRT-PCR. Decreased level of HAGLR was correlated with later stages of the tumor and having poor prognosis [104].

## 6. Conclusion

Cancer research has highlighted the role of exosomes and exosomal materials as new tumor markers and therapeutic targets. Exosomes have a significant potential to serve as a cell-free vaccine and a mediator for drug delivery directed to tumors. Designing exosomes is a fascinating targeted therapy method, and using exosomes as a transporter of drugs has a high potential for specific targeting. Besides the therapeutic approaches, exosomes are an attractive source of materials that provide opportunities to find a new marker with high sensitivity and specificity for screening and diagnosis, especially in lung cancer for which early diagnosis is crucial for successful therapy. Among exosomal materials, lncRNAs are optimal choices for screening because of ease and cost of simple qRT-PCR, besides the fact that lncRNAs are highly tissue specific and are more stable in the circulatory form, particularly if presented in exosomes. lncRNAs are involved in each step of tumorigenesis, metastasis, and resistance to chemotherapy drugs. The specificity of several lncRNAs to malignancy makes them ideal targets as well. While exosomal lncRNAs are in their infancy, the evidence to date is promising for future developments. Aside from the advantages of exosomal lncRNAs, the laboring process of isolating exosomes and lack of established reference genes for lncRNAs are currently limiting. Despite this, continued research should proceed to rectify these issues and further improve lung cancer diagnosis and management.

## Figures and Tables

**Table 1:** Overview of lncRNAs implicated in lung cancer with clinical utility.

lncRNA	Function	Expression profile	Clinical applicant	Cancer subtype	Clinical trial stage	Source of exosome	Year	Reference
MALAT1	Promotion of cell proliferation and migration	Upregulated	Diagnosis	NSCLC	Preclinical	NSCLC patients (#77)	2017	[100]

RP11-838N2.4	Resistance to erlotinib	Upregulated	Diagnosis/target for therapy	NSCLC	Preclinical	NSCLC patients (#78)	2018	[99]

lnc‐MMP2‐2	Increase expression of MMP2	Upregulated	Therapeutic target and predictive marker	Metastatic lung cancer	Preclinical	A549 and HMVEC‐L	2018	[101]

H19	Resistance to gefitinib	Upregulated	Therapeutic target for EGFR^+^	EGFR^+^ NSCLC	Preclinical	HCC827 and HCC827 resistance to gefitinib	2018	[98]

SOX2-OT	Possibly angiogenesis and metastasis	Upregulated	Biomarker diagnostic for LSCC	LSCC	Preclinical	LSCC patient (#75)	2019	[102]

GAS5	Cancer progression	Downregulated	Early stage diagnosis of NSCLC	NSCLC	Preclinical	NSCLC patient (#64)	2019	[97]

GAS5	Angiogenesis	Downregulated	Biomarker	Induced adenocarcinoma	Preclinical	Mice and cell line (A549, H1299, 95D, and 16HBE)	2019	[103]

SLC9A3-AS1	Unknown	Upregulated	Early diagnosis	LSCC	Preclinical	Patients	2019	[96]

PCAT6	Tumorigenesis	Upregulated	Early diagnosis	LSCC	Preclinical	Patients	2019	[96]

HAGLR	Unknown	Downregulated	Prognosis (poor)	NSCLC	Preclinical	NSCLC patients (#40)	2019	[104]

## Data Availability

No data were used to support this study.
